# Experimental Researches on the Ganglionic System

**Published:** 1836-01-01

**Authors:** 


RECHERCHES EXPERIMENTALES SUR LES FONCTIONS DU SYSTEME NERVEUX GANGLIONAIRE ET SUR LEUR APPLICATION A LA PATHOLOGIE. Par *J. L. Brachet,* Medecin de l'Hôtel Dieu, &c. &c. &c. Londres, Dulau & Co. Soho Square.*

EXPERIMENTAL RESEARCHES ON THE FUNCTIONS OF THE GANGLIONIC NERVOUS SYSTEM, AND ON THEIR APPLICATION TO PATHOLOGY. By *J. L. Brachet,* Physician to the Hôtel Dieu, &c. &c. &c. London, Dulau & Co.

WE remember to have heard a distinguished lecturer on physiology once declare that, if the functions of the ganglionic system of nerves were once

* In the 14th volume of this Journal we gave a short notice of this work, taken from a foreign journal, not then having been able to procure the original.

clearly understood, the whole theory of medicine might be contained in a nut-shell. Without attempting to defend or dispute the truth of this assertion, we think we are quite warranted in considering a knowledge of the functions of this important, though mysterious, portion of the nervous system, of the utmost consequence to the practical physician, and that the man who does his utmost to throw light on the subject, is well entitled to the respect of the profession. The various and contradictory opinions which have been from time to time given with respect to the uses of the sympathetic nerve, (some having gone so far as to deny it to be a nerve at all,) sufficiently attest the difficulty of the investigation. To detail in a periodical journal these different opinions, would be uninteresting to our readers, and out of our own province. The every-day works on physiology will give whatever is to be said, and much more than can be proved, on this matter. That this system of nerves is principally concerned in the numerous class of diseases, the neuroses, such as hysteria, hypochondriasis, &c. is the opinion of the first physiologists. The importance of being able to distinguish those diseases seated in the cerebral system of nerves from those which have their origin in the ganglionic system is sufficiently obvious. In a dietetic point of view, an accurate knowledge of the functions of this nerve, presiding, as it does, over those organs by which the important offices of assimilation and nutrition are performed, cannot fail to be peculiarly interesting. However far removed we may be from perfectly understanding the various direct and indirect operations of this system of nerves on the several parts of the animal economy, the consequences of the perversion of these operations are too well known to every medical practitioner—and when it is recollected that these consequences are in a great measure brought on by voluntary indiscretion, and that the greater part of those chronic diseases, which prove at once so distressing to the patient, and so embarrassing to the physician, are purely the results of outrages against the laws of organic life, it will be readily admitted that any advance made towards a knowledge of this system, and of the laws by which it is regulated, must be most desirable, as affording the only glimpse of hope of combatting those obstinate and intractable diseases. The votary of pleasure and the victim of sensuality, those rebels against the laws of organic life, must every day afford to the scientific practitioner painful, though palpable, instances of the dreadful consequences of their rebellion. The illustrious Bichat has been represented to have been the first to shew that the ganglionic nerves are devoid of sensibility; the glutton and sensualist have the melancholy, and exclusive privilege of confuting him; they can boast, and a painful boast it is, that they *feel* they have such a thing as a stomach—but we find we are carried away rather too far from the immediate subject of our analysis; we shall now see what M. Brachet has done to elucidate the physiology and pathology of the great sympathetic.

Our author, after pointing out the importance of a knowledge of physiology, and the only means of attaining correct views of the science, namely,

---

We are now enabled to lay before our readers a full account of M. Brachet's experiments, which, we think, are highly worthy of the reader's attention.—*Eds.*

by studying living man, by animal vivisections, and by cultivating comparative anatomy, adverts to the great obscurity which still hangs over the functions of the ganglionic nervous system. Two nervous systems, he says, are distributed through the animal economy, which diffuse and sustain life in all the organs, are the agents of all sensation and all motion, the dispensers and regulators of the vital actions, the first movers of all the functions. The one system is directly dependent on a principal centre, to which, ultimately, every thing is to be referred. All the experiments made on it produce sensible and appreciable effects, and lead to positive results. Prick a nerve of the cerebral system; the pain of one part, and the disturbance of another, at once point out its uses. Cut this nerve; the paralysis of the organs to which it is distributed complete the discovery. Let us now change the system, and transfer our experiments to the ganglionic nerves—and what a striking difference! The organs are dumb.

To account for this, in some measure, our author refers to the deep situation of the trisplanchnic nerve, which removes it, through nearly its entire extent, from the action of our instruments. In some points only can we reach it, and even there it is not connected with any important organ. The manner in which this nerve penetrates our organs, enables it to elude most of the attempts which might be made on it. It is not one nerve, one cord, but thousands of nerves and of cords, which enter it at once and penetrate it on all sides; so that if we would intercept the communication of an important organ with the ganglionic system, we must, in fact, entirely detach this organ, and deprive it of the materials of nutrition, since it is indispensable to divide the vessels in this way, their coats being traversed by an immense number of nervous filaments. The difficulty of making such experiments has been properly assigned by our author, as the cause of our ignorance of the functions of this part of the nervous system. Since the time of Willis, the most distinguished anatomists have made the great sympathetic the great object of their researches. Among these may be reckoned Vieussens, Winslow, Zinn, Johnstone, Haasse, Reil, and Meckel, who have severally stated opinions on the subject more or less probable. The illustrious Bichat was, however, the first to point out the truth; but, destitute of the aids of experiment and analogy, he was not able to state any thing positive, so that hypothesis has but too frequently usurped the place of fact. M. Broussais has made several attempts to unravel the mystery which hung so long over this department of physiology; but his work on the subject, more distinguished for the brilliant conceptions of a fruitful imagination, than for the results of accurate experiment and rigorous inductions, has by no means attained the desired end. Thus, every thing which has been hitherto done on this subject has rather tended to render it more obscure—certainly it has not removed the difficulty. Thus, says our author, in order to treat the question, we must, after the example of Descartes, forget every thing that has been done or said regarding it—we must recommence with new facts, and strike out a new road for ourselves, in order to attain truth, by studying the functions of the organ in the most simple beings, and then applying them to beings the most compound.

For the sake of order and arrangement in the study of the functions of the nervous system, our author proposes to examine its action: 1st, in organized beings generally considered, and, 2dly, in some particular organs:

the former section of the work being directed to the consideration of the general functions of the great sympathetic, whilst the latter will be confined to its particular or special functions; in the latter division, also, he intends to consider the influence of this system only in some of the organs—those, for instance, in which the influence of the sympathetic is combined with that of the cerebral nerves. He then commences with—

*The General Functions of the Ganglionic Nervous System.* Matter, he says, assumes two forms; it is either brute, inorganic matter, subject to all the physical laws which regulate this vast universe; or else it is living, organized matter, under the dominion of life and of physiological laws. The difference separating these two species of body is immense; on the one hand, death, immobility, unlimited existence, which chance produces and chance destroys; on the other hand, life, sensibility, motion, growth, limited duration, and reproduction, which perpetuates the species. All this is known—it falls under the cognizance of our senses; but what is the primary cause of this difference? What cause puts the line of demarcation between these two great classes of beings? Can it be organization? Yet, however perfect an organized being may be, death strikes it, and it at once becomes immoveable and insensible, and falls under the dominion of physical laws; and still its organization has not changed. It must, then, have had something more, which, the moment before, made it feel, made it move, and enable it to perform those numerous acts which constitute its functions. What is this? Persons tell us it is life, and they then imagine they have explained every thing. But what is life? This happy term, created for the purpose of cloaking our ignorance, evades the question, and does not solve it, no more than the anima, the archæus, the vital principle, &c. which are mere substitutes for it, and which leave us just as far from the truth as ever. Without attempting, says our author, to penetrate, at this period, a mystery which probably will never be penetrated, we should content ourselves with observing facts and their connexion, and thence deducing natural consequences; and we should leave to metaphysicians the sublime researches regarding the nature and origin of metaphysical beings. Whatever this vital principle be, does not now concern us—with its effects alone we have to do: *ubi desinit physicus, ibi incipit medicus,* said Aristotle; an axiom which might be extended into, *ubi desinit medicus, ibi incipit metaphysicus.*

Our author commences with the vegetable kingdom; and, after shewing that these give every sign of possessing the property, or, as he would call it, the function of sensibility, and, going on the acknowledged principle, that every effect must have a cause, and that sensibility is but an effect, lays it down as a necessary consequence, that vegetables are endowed with a nervous system. We shall not follow him in his botanical experiments here, but proceed more directly to what is more immediately connected with the proper subject of the work. Every function, he observes, results from the indispensable combination of two things—organic arrangement, vivified by a nervous system, and the action of stimulants or functional excitants. But, according to the remark of Bichat, all the functions terminate ultimately in sensibility and contractility. This illustrious physiologist, whose creative genius gave a new impulse to the medical sciences, having seen that what established the grand difference between unorganized and organized beings, consisted in the property possessed by the latter of feeling or receiving the impressions of bodies with which they came in contact, and of re-acting on them by a movement, which always originated in the contraction of some part, drew the consequence, that organized beings possessed their peculiar properties just as well as unorganized beings; and to the physical properties, attraction, elasticity, &c. he opposed the vital properties, *sensibility* and *contractility*. Then considering that animals and man in particular, had two kinds of existence, two kinds of lives, by one of which they attained a knowledge of all the beings surrounding them, and were placed in a certain relation to them, whilst by the other they merely acquired nutrition and growth, the latter being an appendage of all organized beings, he distinguished *animal life*, or the life of relation, that life which is exclusively enjoyed by animals, and *organic life*, which is common to all organized bodies; this distinction induced the necessity of another in the vital properties, which were accordingly divided into animal properties, or the properties of relative life, and into organic or nutritive properties. Thus then there were two sensibilities and two contractilities, *animal sensibility* and *organic sensibility; animal contractility and organic contractility*, and the latter was again called *sensible* or *insensible*, according as the phenomena were apparent or not to our senses. By the animal sensibility, the central organ of the life of relation receives all the impressions made on the nerves by the bodies with which they happen to be brought into contact. By animal contractility, this central organ re-acts by its volitions, and determines certain motions which tend to draw us near to, or remove us from, those objects from which it has received sensations. By organic sensibility, which might also be called molecular, each part of the body, organ, or tissue receives the impression, not from the bodies, but from those molecules with which it is brought into contact, without transmitting it to the central organ of the sensations of animal life. By sensible organic contractility, certain organs possess, in their organization, the elements of a motion which is sensible and apparent, but independent of the acts of volition. By insensible organic contractility each tissue being penetrated by the materials which pass through it, and having received from them the local impression by means of the organic sensibility, re-acts on them, molecule by molecule, causing them to undergo in their passage those changes which may be necessary for nutrition, the secretions, exhalations, &c.

In creating the vital properties Bichat, says our author, has rendered them so very interesting, and has presented them under so advantageous an aspect, and has deduced from them such beautiful consequences, that he has well redeemed the errors into which he may have been led. Can we, he says, help thinking as he did, or avoid adopting his sublime ideas regarding physiology and medicine, the properties of organs and tissues, when we read the *Anatomie Generale?* And yet the existence of these vital properties, this child of his imagination, is any thing but proved; or more correctly speaking, Bichat has transformed real functions into mere properties. Modern physiologists have well-nigh adopted these same ideas. Yet in the present state of medicine diseases are no longer entities; the symptoms point out the organ which suffers, but they are nothing of themselves, they have no independent existence. This is the basis of the physiological medicine, the partisans neither do vary, nor can vary on this point. After so explicit a profession of faith, is it not astonishing to see these same enemies of ontology become themselves ontologists? They indignantly proscribe medical entities, whilst they create physiological entities. All their works on physiology recognize vital properties, which they have, it is true, dressed up in divers ways. But whatever be the garb in which they present them, they are still vital properties, the name changes not the thing; let them use the terms *contractility*, *living chemistry*, *vital force*, *irritability*, *excitability*, *tonicity*, &c. let them render them dependent on, or independent of, one another, they are still but vital properties. Now, these properties preside over functions, they act, encrease, diminish, re-act, enjoy, in a word, all the prerogatives of real beings. Yet where do they reside? how do they exist? no one knows; they are imaginary beings, mere entities. Here then is a physiology all together ontological, in as much as all the functions emanate from vital properties, which properties are themselves mere ontological existences. Now, I ask, says our author, what are we to think of a doctrine which proscribes ontology, and which rests on a physiology which is purely ontological? This is a palpable contradiction; and yet it is what every one says, what every one believes and every one repeats. This monstrous union of an ontological physiology with a medicine which is not so, or at least says it is not so, cannot exist without itself destroying the scaffolding which is raised on it. In fact what becomes of this proscription of ontology, when we see ontology itself serve as the basis of medical theory? Is it not an edifice built on sand and undermined at its foundation? In going back to the etiology of diseases, those who are called physiological physicians tell us that they all depend on the alteration of organs, that this alteration depends almost exclusively on inflammation, and they inform us that inflammation is the exaltation of the vital properties, sensibility, contractility, irritability, &c. Here then are factitious beings, whose augmented action, another factitious being, constitutes inflammation the type of almost all diseases. Here then is ontology supporting all the edifice of the physiological doctrine, and yet we say, we know nothing of ontology! or perhaps, we would persuade ourselves that it is sufficient to have proscribed ontology on the one side, in order to have the right of admitting it on the other, and then to make ourselves believe that such is not the case. So then I shall be an ontologist, when, with some, I shall call a fever ataxic, and I am not one, when, with others, I shall recognize a vital force which presides, a living chemistry which works, a contractility which is the base of every thing, which encreases, becomes debilitated, &c. What shall we call ontology, if this be not it? They must reckon very much on our credulity, to think us so ready to adopt every thing without examination. It is evident then that physiology is infected with ontology, and that it keeps medicine in that obscurity and vagueness, from which so many endeavours have been made to rescue it. Vain is the attempt to present the vital properties as mere abstractions, as metaphors. Should we, when the most strict medical language is required, obscure it by metaphorical expressions? Hence, asks our author, does it not become necessary to introduce into physiology the same reform, as M. Broussais has introduced into medicine? would it not be well to make the language of both coincide and harmonize? to overthrow the physiological ontology, by recognizing the entities to which it lends existence and action, as the mere results, effects and acts of organs?

When this truth shall be diffused, then physiological medicine will no longer present that heterogeneous mixture now observable, and all the phenomena of physiology and pathology will be explained, in a manner of themselves, and with the greatest ease. Let us come to the proof of this; 1st. Animal sensibility requires the distribution of nerves through the part, as also their perfect integrity; they are its agents, its instruments; now, we have seen that the action of an organ was its function, thus animal sensibility is the function of the cerebral nervous apparatus. Whether they perceive light, sound, odour, or the tactile properties of bodies, the function is still the same; there is no difference except in the physico-vital organ, which is at its extremities. Why should it be said that there was function in one case, and property in the other? 2dly. Animal contractility is merely action, exclusive of the muscles, wherefore it is their function; 3dly, the same may be said of sensible organic contractility; it is effected by muscles, which differ from the preceding, only in being withdrawn from the influence of the will; their action however still continues the same, and this action is a function, since it has particular organs, and cannot be performed by any others; 4thly, Organic sensibility exists only in virtue of a nervous system, which, having only to preside over vital functions had no necessity for a single centre to which it should transmit its sensations; but though not perceived by the encephalic organ, this sensation still exists, as might be proved by a thousand experiments, if necessary. Who may not see liquids taken into the stomach absorbed with greater or less rapidity, according to the mode of sensation produced by them on the digestive passages? Who does not see these same liquids when absorbed, excite such or such a secreting or excreting organ in preference to any other, become changed into a recrementitious liquid, or be again carried out of the system by sweat or by urine? This sensibility being itself an action, since it is performed by a nervous system, namely the ganglionic, it is also a function, and a function too of this same system.

We must say, before we go farther, that the charge of begging the question, or more strictly speaking, of assuming too much, may be brought against the author in this place. He wants to show that the sympathetic nerve, which some very distinguished physiologists, and among them M. Majendie, deny to be a nerve at all, constitutes an independent nervous system. To prove this, he mentions several actions which take place in the animal economy; these he assumes to be performed by the sympathetic nerve; and then he infers—but we shall say no more here, until we come to the second division of the work, wherein, if he gives us any thing like a *proof* of the agency of the ganglionic nerves in these several phenomena that are going on in the system, we shall most willingly either retract, or modify the charge, which we think, as far as our author has yet gone, might be fairly brought against him.

Our author seems determined in exploding from medical language altogether the term vital properties, since, he says, what has been so called, is the constant result of the action of an organ, is in fact a function. This mode of viewing the matter he thinks, will by rectifying the ideas, rectify also the language of physiology. Thus instead of speaking of the action of an excitant on the animal sensibility of an organ, we shall say that it acts on the cerebral nerves of this organ, consequently on a function and on its organ; instead of saying that touching the uvula excites the organic contractility of the stomach, we should say that it excites its muscular layer. In vain, then, have the terms tonicity, irritability, &c. been introduced to designate the organic vital properties—such substitutes are any thing but happy.

Our author next anticipates an objection which he apprehends may be started against the reform he is so anxious to introduce into medical nomenclature. It may be stated, he says, that it matters very little whether sensibility be a property or a function, an agent, or an act of an organ; that the functions are not at all changed, and that they still remain the same. To this objection he answers, that he will apply the same reasoning to the physiological doctrine, by putting it for a moment in the place of physiology, and then say that the reform introduced by M. Broussais is altogether useless; that diseases are still the same, whether they be regarded as the effect or cause of the phenomena, and that the gastro-enteritis, whilst no longer an essential fever, has not changed its character, but still is and always will be the same disease, &c. Will not, he says, every physiological physician put down such reasoning as false and inconclusive? One of two things, either ontology has injured the progress of science, or it has not. If it has, as every one must admit, it should be attacked even to its very entrenchments, and pursued and hunted down in physiology as has been done in pathology. What M. Broussais has done for medicine, let us, says our author, do for physiology, and let not silly prejudice, and a lingering attachment to old studies and old opinions, retard us; let us lay aside all false shame, and proclaim openly and with confidence, that physiology shall no longer be an ontological being, no more than pathology. Then we shall cease to see a property, encreased or diminished, become a disease; we shall cease also to direct against this property, this metaphysical entity, physical or material medicaments. We shall see in the healthy body tissues, organs, and fluids: we shall find diseases to consist in the alteration of these principles; and we shall no longer set about treating mere properties, but organs, tissues, or altered fluids. Organised beings, he says, are divided into vegetable and animal. We know that the former discharge their vital or organic functions under the influence of ganglionic nerves. By analogy then we may conclude, that Nature, always uniform in her processes, has not created different organs for the same functions. Consequently, absorption, the capillary circulation, nutrition, the different secretions and exhalations, take place in animals, as in vegetables, under the influence of the ganglionic nervous system.

(Assuming the analogy in this case seems to us something very like assuming the entire question at issue. But we shall not interrupt our author.) Such, he says, was the opinion of Bichat, who gave this system the name of the nervous system of organic life; such was also the opinion of several other eminent physiologists, ancient and modern; but Bichat, unwilling to extend the action of organs farther than he could trace them with the scalpel, and not having carried his researches regarding the great sympathetic far enough, limited its action almost to the great visceral functions, and supplied the defect by the organic vital properties, wherever he could see no nerve. The difficulties which then existed no longer exist; the patience and industry of anatomists have surmounted them; and the trisplanchnic nerve has been traced into the remotest regions, on the arteries which it accompanies, forming a plexiform network, many branches of which assume a gangliform appearance, regenerate and recruit the nerve and perpetuate the chain; as, without this successive renewal, it would have been impossible that a fine filament could supply an entire limb. Thus, in animals, the ganglionic nervous system differs from that in vegetables neither in its anatomical arrangement, nor in its functions. In both we find a long nervous cord, frequently interrupted by bulgings or ganglions, which are the centres, the terminations, or origins of each nerve. Those ganglions are the preservers, the regenerators of vital action, since by destroying them life is abolished in the parts to which the nerves supplied by them are distributed; then the vegetable deprived of them is no longer reproduced, whilst the smallest portion of a plant may become an entire plant, if it possess a single nervous centre. In both we find a distribution by means of filaments, which are not exhausted and extinguished by supplying the several organs, but which are multiplied, and, in a manner, reproduced, in the midst of a small body, a small central ganglion, which is placed either in the course of the filaments, or in the substance of the organs.

What we have to advance, regarding the functions of the great sympathetic, would end here, says our author, if the animal economy were as simple as the vegetable, or if these two orders of functions connected with the life of relation and the life of nutrition were entirely isolated. But animals are far removed from this functional simplicity, and it becomes extremely difficult to establish a line of demarcation between their different functions. They form a chain so closely connected that they all depend on each other, so that it is impossible to alter one without altering the rest. However, all the functions do not exist in all animals, nor even after the same manner. They follow a progression easily remarked, so that according as new organs become developed, new functions and new complications result from them. Besides, in vegetables, the nutritive functions are alone and are performed there by the most simple apparatus; whilst in animals the combination of the nutritive functions with the functions of relation has produced immense changes even in the organs of nutritive life, and has induced the necessity of more complicated, as well as of additional functions. Thus digestion, the circulation, respiration, are no doubt nutritive functions; but they are foreign to the vegetable, because the latter, destitute of animal life and fixed to the soil, must find around it the materials of these functions. In animals again, the mode of existence being different, they must be modelled on this new existence, and be adapted to its nature. Thence this admixture of actions and of functions which prevents us from perceiving the line of demarcation; thence this influence of one life upon the other, or rather of the nutritive functions on the cerebral functions, and vice versa.

Here our author describes the portions of nervous system allotted to the different orders of organized being, commencing with vegetables, zoophytes, &c. till he arrives at vertebrated animals; and, regarding the long series of these, he says, that whatever be the great differences which separate them, and which constitute so many distinct orders, the nervous system preserves the same disposition in them all; it accommodates itself to the modifications of the different functional apparatuses. In all we find, 1st, a brain enclosed in a bony case, sending off from itself, or its spinal prolongation, the nerves of the senses and those of the organs of motion; 2dly, a ganglionic nervous apparatus, situate outside the spinal cavity, and placed before it, where it extends in two uninterrupted cords, and which, from each ganglion, send a crowd of filaments to the splanchnic organs and to the vessels; 3dly, a nerve setting out from the brain, and distributed, conjointly with the preceding, to the internal organs, to the viscera of nutritive life. According as the animals are raised to a more distinguished rank by their organization and their faculties, the brain acquires more development, new parts are added to the parts already existing; so that we may in general judge, *à priori,* and by mere inspection, of the degree of intelligence of the animal, to which the brain under examination may belong. With respect to size, see the immense difference subsisting between the brain of the whale and the brain of man, and you will not be astonished at the superiority of the faculties of the one above those of the other. It is by this apparatus that animals are what they are; it is by it that the one is endowed with surprising strength, and that the other makes up for the want of strength by ruse and agility; that one shews uncommon intelligence, whilst another charms by its musical talent. Suppress the brain, and all is inert and devoid of motion; no perception, no reflection, you have in fact a mere vegetable.

In order to shew that this suppression of the brain is not a mere supposition, our author mentions a very curious case, detailed in Graefe and Walther's Journal, by Professor Meyer of Berlin, of the total absence of all the cerebral nervous system;—the history of the case is very complete, and more rich in anatomical details than any we possess on heteradelphous beings. On the parasitic attachment of that which forms the subject of it, no part of the cerebro-spinal apparatus could be discovered. The sciatic, crural, and obturator nerves were entirely wanting. Neither could any nervous filaments be observed entering the muscles. All that could be found was a small filament accompanying the crural artery, and which appeared to come from the renal plexus; but with respect to this plexus, it could be distinctly recognized, as also the mesenteric plexus; they were both provided with ganglions. This heteradelphous fœtus must then have grown solely under the influence of the ganglionic nervous system, this being the only system it possessed. It was in fact a real vegetable.

We have now seen, says our author, the ganglionic nervous system serve as the sole origin of the nerves of vegetables, preside also over their functions, without forming any single centre, or *sensorium commune.* We have seen the system of ganglions, in the lower classes of animals, combine with a new system, having a common centre, which was so much the less extensive, and less isolated, as the functions were as yet but instinctive, and confined merely to the wants necessary to the support of life. We have seen these rudiments of brain assume more development, become more isolated, and form more and more a separate and independent system, according as the relations of the animal become multiplied, and according as it no longer lived solely within itself, and established more extensive relations with surrounding beings. The separation, however, is never complete; numerous communications always connect these two orders of nerves; but the more we ascend in the class of beings, the more the cerebro-spinal attains the superiority. Remove the encephalic and spinal mass, and the large nerves which derive their origin from them, compare them to the trifling assemblage of the ganglions and their nerves, and you will soon conclude that man is called to far nobler functions than to vegetate, grow, and be nourished—the cerebral system, the organ of intelligence, being so superior to the ganglionic system. In vegetables, it was easy to appreciate the functions of the ganglionic nervous system, since it is sole, and presides solely over their functions. In the lower animals, it was easy to appreciate the action of their ganglionic system, and its influence over these same functions; the same effect recognizes the same cause. But, according as the animal has become more perfect, and its functions more complicated, the result is, a combination of action between the cerebro-spinal and ganglionic systems, which was indispensable.

It is this combined influence of the two systems on the assimilating functions, that will form the subject of the second section, and which our author intends to examine in the principal organs of the animal economy; he calls it combined influence, because the anastomoses which exist between the two systems, and which establish numerous relations between them, do not render them dependent on each other. Thus, whilst the one is at rest or in sleep, the other continues to act, and acquires even a well-marked preponderance—whilst the one has ceased to live, the other still manifests its vital influence, whilst the one exercises and suspends its functions momentarily and at intervals, the other exercises them continuously, and without intermission. He now proceeds to the SECOND SECTION, in which he considers

*The particular or special Functions of the Ganglionic Nervous System*—by which is meant the action of the great sympathetic on each determinate organ. He premises that it is his intention to consider only the principal functions, those, more particularly, which are peculiar to the animal, and are wanting in the vegetable. He intends, in fact, to limit his enquiries into the action of the great sympathetic—1st, in the alternate contraction and dilatation of the heart—2dly, in the different acts of respiration—3dly, in the action of the stomach on aliments—4thly, in the intestines—5thly, in the bladder—6thly, in the organs of generation—7thly, on the secretions—8thly, in the sympathies—9thly, on vision—10thly, on the passions. Each of these subjects he intends to consider in a separate chapter.

And, first, with respect to the influence of the ganglionic system of nerves on the heart's action:—Of all the organs, says our author, the heart is that whose action is most simple. Being composed merely of muscular fibres, its functions differ in no respect from those of other muscles. Regarding the heart's contraction, the field was for a long time open, for want of positive experiments. Thus, some fallacious experiments induced Haller and his school to make irritability a property inherent in the fibres of the heart, and made Fontana say, that the nerves of the heart were of no use whatever, whilst Behrends and Soemmering denied that this organ received any nerves, in which latter opinion, several very distinguished physiologists joined. At the present day, since the splendid researches of Scarpa, no one any longer doubts but that the heart receives nerves. But what are they? and in what manner do they influence its movements?

The heart receives nerves, and a considerable number of them. The pneumo-gastric nerves and the cervical ganglions of the great sympathetic furnish the filaments, which, by combining, go to form the great cardiac plexus, and thence are distributed to several points of the heart, subdividing into several secondary plexuses. The nerves supplied by the eighth pair, are much less numerous than those which come from the ganglions. Nature has done nothing in vain; thus these nerves, from whatever part they derive their origin, must have an influence. The question now is, to determine this influence, and to what nervous system it belongs. To attain the solution of this question, it becomes necessary to act, on the one hand, on the cerebro-spinal system; on the other hand, on the ganglionic system.

To determine the influence of the former, it would appear to be quite sufficient to isolate the heart, by dividing the par vagum, the only mode of communication existing between them. Our author, however, in order to attain greater certainty, and to leave no room for objection, directed his experiments, first, to the encephalon—secondly, to the spinal marrow—thirdly, to the pneumo-gastric nerve—and, 4thly, to the ganglionic nerves.

*Influence of the Brain on the Heart.* If the heart contracts only in virtue of the more or less direct influence which it receives from the brain, the destruction of the latter, or the abolition of its functions, must necessarily abolish or paralyse the heart's movements. If, on the contrary, the contractions of this organ are independent of the influence of the brain, it will be in vain to destroy or remove the latter—they will continue.

*Exper.* 1. I removed in succession, and by slices, the brain of a rabbit, three months old. The removal of the first portions produced no appreciable effect on the movements of the heart. According as I carried the instrument over the deepest layers, the heart became very much disturbed, and its contractions often irregular. I touched neither the annular protuberance nor the cerebellum. The animal lived eighty minutes, during which the heart ceased not to beat with regularity, though with more rapidity than natural. The left thigh having been cut at the end of an hour, there was a jet of arterial blood jerked forth feebly, but regularly.

*Exp.* 2. From a rabbit, of the same age, I removed the entire of the brain. At the moment of the removal, the heart became agitated, in some measure, convulsively; after leaving the animal to rest for two minntes, the contractions of the heart became regular. I removed progressively the entire cerebellum, proceeding from above downwards. The heart was agitated irregularly during the entire time of the operation. Two minutes after the operation terminated, the heart continued to beat with rapidity, but with sufficient regularity to keep up the circulation for twenty-three minutes. The annular protuberance was left untouched. The amputation of a fore-paw caused arterial blood to flow some minutes before death.

*Exp.* 3. On a third rabbit, which was better than three years old, I removed the brain and cerebellum as rapidly as I could: the same disturbance in the heart's action took place. I removed a portion of the medulla oblongata, the disturbance was redoubled. I removed the entire annular protuberance; and the heart, after some irregular contractions, ceased to beat, and the animal died.

*Exp.* 4. I repeated the same experiment on a rabbit ten days old. After the first impression of reaction caused by the instrument in each part of the operation, the heart continued to beat regularly for twenty-five minutes.

The artery of a fore-foot furnished, several times, a jet of vermillion blood.

*Exp.* 5. I removed from a cabiai of eight days old the brain, cerebellum, and annular protuberance. The disturbance of the heart and the irregularity of its contractions were extreme; I thought the animal was dead. However its calm was re-established. I then removed, as deeply as I could, the extremity of the medulla oblongata. Some irregular movements took place in the heart; and the animal died in less than two minutes.

*Exp.* 6. The preceding experiment was too important not to be several times repeated. It might be conclusive, since death had followed closely on the removal of the part of the encephalon, in which the pneumogastric nerves arise. In consequence of this I removed the brain, the cerebellum, and the medulla oblongata, as far as below the origin of these two nerves, taking all the precautions possible that death might not be attributed to any other cause. This prevented not the animal from perishing in less than three minutes. I obtained the same results for several cabiais of different ages.

Notwithstanding my desire, says the author, to attain my end, I could not satisfy myself that I had succeeded. I possessed too many facts which confirmed me in the idea, that death must recognize another cause, and that the heart should contract notwithstanding this removal. At last I perceived that the stoppage of the larynx was the true cause of death, and that the animal died of asphyxia. Henceforward it was easy to obviate this inconvenience in my subsequent experiments.

*Exp.* 7. I took a rabbit two months old, made an incision in the trachea below the larynx, and placed a canula there to keep up the respiration. I proceeded to remove the brain entirely as far as the origin of the spinal marrow. I saw the heart, which at first was agitated rather irregularly, contract for upwards of fifteen minutes, after which I cut off a fore-paw, and I saw the blood gush forth.

It has been remarked by all physiologists that these experiments succeed much better when made on young animals, in which the brain as yet exercises but inconsiderable influence. The natural consequence to be deduced from these experiments is, that *the brain exercises no direct influence on the heart.*

This consequence, he says, has been repeatedly confirmed by pathology. In certain wounds of the head the brain has been entirely removed, and the heart continued to beat for some hours. Some experiments contained in Hufeland's journal also confirm it. Six high-way robbers had been decapitated near Marburg;—the bodies of them all were opened some minutes after decapitation. The heart was observed to contract and dilate alternately with considerable strength, so as to produce regular pulsations. The movements continued to decrease for nearly half an hour. Irritation of a filament of the great sympathetic re-animated them for a moment: irritation of the spinal marrow produced no effect on the heart, whilst it caused the muscles of the trunk to contract. Acephalous fœtuses prove the same thing.

What, says our author, is more adapted to demonstrate this independence of the heart, than a glance at what takes place in paralysis, epilepsy, in the effects of opium, of narcotic and alcoholic drinks? An effusion of blood, serum or pus, tubercles, an accidental ossification, compress the brain, paralyse its action, and with it all the sensorial or motor organs, and still the circulation continues. He next considers the—

*Influence of the Spinal Marrow on the Heart.* The anatomical, physiological, and pathological study of the spinal cord had been neglected very much till latter times, notwithstanding some very ingenious views of Willis. To Legallois we are indebted for having directed the attention of physiologists to this part of the system, and for having led the way to new discoveries. Our author tells us, that he was present at most of the experiments made by him at the school of medicine, and that he was seduced by them with every one else. He thought with him that the principle of the heart's contractions resided in the spinal marrow, and that this part of the cerebral system was the true seat of the vital principle, since its destruction destroyed life by paralysing the organ of the circulation. The enthusiasm of novelty having given way to reflection, some of the experiments of Legallois inspired him with doubts, and induced him to resume the subject. The conclusion to which his experiments led him to come was, that the contractions of the heart, which keep up the circulation, are entirely independent of the spinal marrow. His experiments fully satisfied him that the heart might contract and the circulation continue after the complete destruction of the spinal marrow. He cautions his readers not to misunderstand him in his use of the term contractions; by it he means those regular normal contractions which keep up the circulation. This complete independence of the heart's contractions form an exception to a general law, which our author tells us he had been led to deduce from the experiments of Legallois, namely, that wherever muscular fibres did contract they did so under the influence of cerebral nerves. This independence of the heart's action, the fact of it alone, which is the principal mover of the circulation, being exempt from the control of the cerebro-spinal influence, he considers an additional proof in favour of the individual existence of the nutritive or ganglionic life. Every where, says our author, the muscular fibre owes the regularity of its contractions to the cerebral influence—the fibre of the heart is alone exempt from it: this fibre recognizes another influence, it is then totally unconnected with cerebral life. The stomach and intestines, it will be said, appertain to organic life, and yet the contraction of their fibres is under the influence of the cerebral nerves. I deny not this fact, as I have established the former; but I shall observe that these organs, as well as the bladder and uterus, are not organs essential to organic life; that they are but organs of circumstance, adapted to the convenience of the animal; the proof of which is, that they are wanting in the greater number of living beings, whilst the circulatory apparatus is not wanting in any one. The organs, created for the animal, must therefore depend on the organ which essentially constitutes the animal, namely, the brain; whilst the heart is entirely an organ of nutritive life; the functions of this life are performed solely by the circulation; digestion, the urinary secretion, and uterine conception, are not indispensable to it, these being wanting in several organized beings.

As in most of the experiments of Legallois, and in all those made on animals of a certain age, we see the heart suspend its movements almost instantaneously, it may be asked, whether, such being the case, it is true that this organ is independent of the cerebro-spinal system, and one may be disposed to question that isolated state of the heart just now established. This fact is true, says our author ; but as one fact can never destroy another, and their apparent contradiction is owing to there being between them something intermediate which escapes us, it does not in any respect change either our experiments, or the consequences drawn from them. It only proves the association and close relations which subsist among all organs. It proves more particularly that the farther we remove from the first period of life, this association becomes more intimate, it being no longer possible to destroy it. The more the cerebro-spinal system is exercised, the greater its influence becomes, the more the two lives are united and combined, the less possible is it to separate them : they no longer form but one, though each organ preserves its functions.

The author, in further confirmation of his conclusions, appeals to morbid anatomy. He mentions a case from Morgagni of a child born at the full time, without either cerebrum, cerebellum, or spinal marrow—nutrition had gone on perfectly well ; the child was well-formed, and free from all stench. Several other cases he also cites of a similar nature. He concludes by stating that his experiments, pathological facts, and the study of the development of the cerebro-spinal organ have furnished him with the most convincing proofs, that the heart derives not the principle of its action from the spinal cord and that it is perfectly independent of it. He next proceeds to consider the

*Influence of the Pneumo-Gastric Nerves on the Heart.* We have already said, that the heart receives nerves from two sources, from the great sympathetic by the cardiac nerves, and from the brain by the pneumo-gastric nerves. These latter nerves might incline one to think that, being the medium of communication between the brain and heart, the cerebral influence must be transmitted through them to this latter organ. Several experiments were undertaken by some of the old physiologists, both by tying, and dividing these nerves, in order to ascertain their influence on the heart's action. Willis was among the first of these experimenters. He divided the par vagum, and from the disturbance caused in the circulation by the operation, and the subsequent death of the animal, on which he experimented, he concluded that to these nerves the heart owed its circulatory powers. After the re-organization of the medical schools in France, the most distinguished physiologists recommended the experiments on the pneumo-gastric nerves; they observed that their division produced a perceptible, though merely a temporary, disturbance, in the circulation; but that this disturbance never arrested it, and that it never was the occasion of death, which was caused by the more serious disturbance of other functions. On recapitulating all that has been said regarding the influence of the cerebro-spinal nervous system on the heart's action, we shall see that the system is not necessary to keep up the circulation, since 1st, the brain may be entirely removed; 2ndly, the head may be cut off; 3dly, the spinal marrow may be destroyed artificially or be naturally wanting; and lastly, the nervi vagi may be tied, cut and destroyed, without this function being abolished. However, as we have seen every experiment produce a disturbance in the movements of the heart, we cannot refuse to recognize a sort of *consent* and necessary union between the principal organs of the economy. The filaments of the pneumogastric nerve which join the cardiac plexus, explain the re-action of the encephalon on the heart. The numerous filaments which establish the communication between the cervical ganglions and the spinal marrow, explain the re-action of the latter. These two modes of communication, on the one hand, of the brain with the heart, on the other, of the spinal marrow with the ganglions which give out the cardiac nerves, account for the numerous sympathies which subsist between these two apparatuses, in health as well as in disease. There is no well-informed physician who does not appreciate the multiplied phenomena resulting from this harmony. Though the cerebral influence on the heart may not be absolute, it still exists, and is very extensive; it is it which performs the principal part in the numerous passive sympathies of the heart. An organ suffers, the heart is sympathetically disturbed; the thing takes place in this way: the cerebral nerves of the affected part are irritated and transmit the irritation to the brain, this re-acts on the heart, and thence the circulation is accelerated. This is so true, that if the communication between the brain and heart be interrupted, there is no longer any re-action, the heart is passive amid the greatest excitement.

The author here introduces two experiments on dogs; in the first of which he divided the two pneumo-gastric nerves, in a dog of six months old, with the usual precautions to prevent suffocation. An hour after the animal was perfectly tranquil and the heart beat regularly. He irritated the wound, and pulled the upper extremity of the nerve; at each pull the animal manifested signs of pain, the heart remained passive, and its movements were unchanged. Some minutes after he opened the cranium and lacerated the brain; the animal fell into a state of stupor without the heart being affected; he then carried the instrument towards the medulla oblongata, when different convulsive movements took place, the heart still continuing passive; on plunging the style deep into the vertebral canal through the occipital foramen, the respiration was arrested, some irregular movements of the heart were observed and the animal died. In the next experiment he practised the same treatment on a dog of the same age and size, except that in this case he left the nervi vagi untouched, though the same wounds were made in the neck as in the preceding experiment. After one hour's rest, he irritated the wounds of the neck in different ways and soon observed the heart's contractions to become very much disturbed and hurried. After a half hour's rest calm was re-established, the cranium was opened, and several portions of the brain were removed; a needle was then carried as far as the medulla oblongata: the heart became very much disturbed, and its action very irregular. The two pneumo-gastric nerves were then divided, the disturbance of the circulation continued; on establishing the artificial respiration, the heart gradually contracted with more regularity. A needle was carried in every direction and over every part of the medulla oblongata without any effect, the heart continued passive, but ceased to contract as soon as the needle was plunged into the spinal canal.

From these two experiments our author infers that the brain is the intermediate agent in a number of sympathies, that it is at first influenced itself, and then re-acts. The passiveness of the heart when the medium of communication between this viscus and the brain was destroyed, and the acceleration of its contractions, when the pneumo-gastric nerves remained untouched, sufficiently establish this active part taken by the brain in sympathies.—The pneumo-gastric nerves not only serve to establish the re-action of the brain on the heart, they also serve to transmit the re-actions of the heart on the brain; they maintain a reciprocal influence between these two organs. It is by them that the brain perceives the pain of carditis and pericarditis. It is by them also that that very painful sense of strangulation is occasioned, that real convulsion of the larynx, produced by the recurrent nerves. It is by the cardiac nerves, on the contrary, that the pain is carried to the back, to the lower cervical ganglions, where they come to derive branches of communication with the spinal marrow. It is by these same nerves that the spinal marrow is so deeply affected as to be almost paralysed in its re-action on the respiratory muscles, whence result those distressing dyspnœas so frequently met in diseases of the heart.

*Influence of the Great Sympathetic on the Heart.* As the heart does not contract under the influence of the cerebro-spinal system, it becomes necessary to seek the source of its movements elsewhere than in the brain, cerebellum and spinal chord: or else, after the example of the Hallerian School, are we to find the principle of its contractions in its own organization, and are we to present its fibre as the type of irritability? But, if the heart was not subjected to the nervous influence, why should it receive nerves? Since Scarpa's work, can it be said, with Soemmering and Behrends, that the cardiac nerves do not penetrate into the muscular fibres of the heart? or, with Fontana, that their use is not known? Does not Haller contradict himself, when, after having spoken of anencephalous fœtuses, which might have furnished him so powerful an argument in favour of irritability, he adds: *Plerisque medullæ spinalis etiam fuit tantum, quantum sufficere poterat, ut cordis motus superesset.* We have, says our author, already seen that the pneumo-gastric nerves, the only cerebral nerves which go to the heart, have no direct influence on its contractions, since the circulation may continue for several days after they have been divided. In reasoning thus by remotion (par voie d'exclusion) it might be supposed that the cardiac nerves, which come from the ganglionic system, were the only nerves remaining to carry to the heart the nervous influence, which directs its regular contractions. Since the opinion expressed by Winslow regarding the attributes of the great sympathetic, an opinion adopted and sanctioned by the illustrious Bichat, the ganglionic nervous system, is destined to the functions of the organs of nutrition. Now, the heart being one of the principal organs, the centre in some degree of this interior life of nutrition, must naturally be immediately dependent on the organic nervous system.

However seducing such a mode of reasoning may be, it holds out but mere probabilities, and, as all these probabilities together can never become equivalent to a proof, nor establish a truth, it may be objected that the nervous system of the ganglions exercises no more influence on the contractions of the heart, than the cerebral system, this influence not being attested by any experiment. Our author feeling the full force of this objection and acknowledging its importance, set himself in search of some means of proving this action of the ganglionic nerves on the heart. Two means of accomplishing this presented themselves to him; either to destroy all the ganglions which supply the cardiac nerves, or to come near the heart and divide these combined nerves. After several fruitless experiments on dogs, the failure arising from the hemorrhage which ensued, he succeeded, after placing a ligature on the two subclavians, in isolating on both sides the inferior cervical ganglions; he then divided all the nervous filaments proceeding from them. Instantly the heart, after a few irregular contractions, ceased all its movements, and the circulation stopped; the exposed carotids no longer beat; and when opened, they yielded but a few drops of blood. He repeated the experiment on another dog, and with the same result. Here, then, as the division of the ganglionic nerves going to the heart paralysed this organ, the obvious conclusion was, that these nerves were the agents of the heart's contractility. In another experiment made on several dogs, he says he saw the movements of the heart, which were at first irregular, after their first disturbance resume their calm state in some measure, remain more accelerated, but sufficiently regular to restore the circulation. I then, he says, sought what could be the cause of this difference in the result, and I attributed it to this circumstance, that the upper cardiac nerve sufficed to restore life to the cardiac plexus, and to keep up the regularity of those contractions so necessary to the performance of the circulation. So that, to be sure of success, it became necessary to divide at one and the same time the two upper cardiac nerves. A reflection here suggested itself. If, said I, it be true that the ganglions are independent of each other, and that each forms a nervous centre peculiar to the nerves which go from it, I shall to no purpose isolate the heart from all the cervical and ganglionic nerves which go to it. It will continue its contractions since this organ will preserve intact its ganglion, or upper cardiac plexus.

If this be the case, what we call the cardiac nerves do not deserve this name; they are but mere filaments of communication between the cardiac ganglion and surrounding ganglions; they are destined to keep up that harmony of functions so necessary to the union and consent of their performance, and to the production of the numerous sympathies of this organ; but they are not the agents of the heart's impulsion. In this sense, the cardiac ganglion resembles the ophthalmic ganglion, which has no necessity to communicate with the other ganglions to exercise its influence on the organ of vision. This mode of viewing the heart's action, as independent of all foreign influence, rendered new researches necessary. It was no longer on the brain, cerebellum, spinal cord, nor on the cervical ganglions, that we must act, but nearer to the heart, on the nervous centre which directly supplied it with its nerves; in a word, on the cardiac ganglion. The position of this ganglion made me (says our author) despair of succeeding, and I could not help admiring the wisdom of Nature in having withdrawn from the action of foreign bodies what is probably the most important organ of life, and particularly in having secured, more than any other, the ganglion from which it derives the principle of its contraction, by so placing it, that it could not be reached but by destroying the most essential organs, which are ranged around it as if to protect it. It became necessary, however, to get at this ganglion; otherwise every thing was vague and uncertain with respect to the principle of the heart's action. After several attempts, says our author, I at length succeeded as follows:

"On a young dog I placed a double ligature as near as I could to the origin of the left subclavian. I divided the artery between the two ligatures

I exposed the cardiac nerves, traced them into the chest, turned back the first rib, by disarticulating its sternal extremity, and arrived by degrees at the cardiac plexus. When I thought I had it isolated, I carried the scissors into the wound and divided the plexus. The circulation, which but an instant before went on perfectly well, was at once arrested; the heart ceased to contract, the animal became convulsed, and died. I opened the chest, and the examination of the parts satisfied me that the cardiac ganglion was perfectly divided, so as to isolate the nervous filaments which proceed from it to form the other cardiac plexuses. The animal had lost but little blood, and the pulmonary vessels were gorged. Hence the death of the animal was the immediate and inevitable consequence of the division of the cardiac ganglion." In another experiment on a terrier, in which he adopted the same course as in the preceding, the contractions of the heart, though irregular, continued and kept up the circulation; they continually became weaker, and after forty minutes the animal was dead. This experiment overturned the ideas suggested by the preceding. The cause of the difference, however, he found to be this, that the ganglion, from its form, was but partially divided; so that what remained not cut was sufficient to keep up the nervous influence; in the same manner as the brain may be partially destroyed, and still continue some of its functions. The animal, however, did not live long.

The inductions to which the author was led by reasoning, were thus confirmed by experiment. So that it is not merely because the brain, cerebellum, and spinal marrow are not necessary to the regular contractions of the heart, that we are to conclude that the great sympathetic is the nervous system which presides over these contractions; it is because experiment has proved to us that the circulation requires that system in its integral and perfect state, in order to be performed freely; and that every time its communications with the agent of impulsion were interrupted, the heart ceased to contract, and the circulation was abolished.

We have now followed our author in his experiments to prove the influence of the ganglionic system of nerves on the heart's action. In our next number we shall continue the analysis through the remaining part of the work, in which he endeavours to establish the influence of this system over the performance of the other vital functions.

